# Rickettsial Seroepidemiology among Farm Workers, Tianjin, People’s Republic of China 

**DOI:** 10.3201/eid1406.071502

**Published:** 2008-06

**Authors:** Lijuan Zhang, Ailan Shan, Bobby Mathew, Jieying Yin, Xiuping Fu, Jingshan Zhang, Jie Lu, Jianguo Xu, J. Stephen Dumler

**Affiliations:** * National Institute of Communicable Disease Control and Prevention, Beijing, People’s Republic of China; † Tianjin Municipal Center for Disease Control and Prevention, Tianjin, People’s Republic of China; ‡ Johns Hopkins Medical Institutions, Baltimore, Maryland, USA

**Keywords:** Anaplasma phagocytophilum, Ehrlichia chaffeensis, Bartonella henselae, Coxiella burnetii, Rickettsia typhi, seroprevalence, farm workers, China, dispatch

## Abstract

High seroprevalence rates for *Anaplasma phagocytophilum* (8.8%), *Coxiella burnetii* (6.4%), *Bartonella henselae* (9.6%), and *Rickettsia typhi* (4.1%) in 365 farm workers near Tianjin, People’s Republic of China, suggest that human infections with these zoonotic bacteria are frequent and largely unrecognized. Demographic features of seropositive persons suggest distinct epidemiology, ecology, and risks.

Human granulocytic anaplasmosis and monocytic ehrlichiosis are emerging tick-borne rickettsial diseases (*1*,*2*). Like other rickettsial infections, these diseases are distributed worldwide but are predominantly reported in the United States or Europe (*2*). Despite evidence of *Anaplasma phagocytophilum* and *Ehrlichia chaffeensis* in ticks and rodents in the People’s Republic of China (*3*–*7*), few investigations have been conducted. A pilot survey in Jiansu, Zhejiang, Shandong, and Hubei Provinces during 2004–2005 and an unusual cluster of cases in Anhui Province in 2006 identified human granulocytic anaplasmosis. As a result, a seroepidemiologic investigation was undertaken to assess exposure to *A*. *phagocytophilum*, *E*. *chaffeensis*, *Bartonella henselae*, *Coxiella burnetii*, and *Rickettsia typhi* among persons on 8 farms in 7 districts and rural counties near Tianjin, China.

## The Study

Tianjin is located in the northeastern part of the Huabei plains in China. It has a temperate continental climate. The surrounding area comprises 13 districts and 5 rural counties. The study was conducted on 8 farms in 7 districts (Beichen, Dongli, Dagang, Xiqing, Jinnan, Tanggu, and Hangu) and Ninghe County.

*A*. *phagocytophilum* (Webster strain) and *E*. *chaffeensis* (Arkansas strain) antigens were prepared from infected HL-60 and DH82 cells, respectively. The *E. chaffeensis* strain used was provided by the US Centers for Disease Control and Prevention (Atlanta, GA, USA), and the *R*. *typhi*, *C*. *burnetii*, and *B*. *henselae* strains and antigens used were provided by the World Health Organization Collaborating Center for Rickettsial Diseases (Marseille, France).

From May through July 2006, 365 healthy farm workers in close contact with domestic animals, vectors, or rodents were included in the analysis. A questionnaire was used to record demographic data, sex, age, occupation, length of service, and farm animal contact. The study was reviewed and approved by the Tianjin Institutional Review Board.

Serum samples were obtained from the 365 participants. Tests for antibodies to *A*. *phagocytophilum*, *E*. *chaffeensis*, *B*. *henselae*, *C*. *burnetii*, and *R*. *typhi* were performed on 220 samples at the National Institute of Communicable Disease Control and Prevention Laboratory. Serum samples from all 365 workers were separately tested for antibodies to *A*. *phagocytophilum* at Johns Hopkins University School of Medicine.

Serum samples were diluted 1:80 in phosphate-buffered saline, and 25 μL was placed on antigen slides and incubated for 60 min. Slides were washed, incubated with fluorescein isothiocyanate–conjugated goat antihuman immunoglobulin (Ig) (IgM plus IgG; Sigma, St. Louis, MO, USA) for 60 min at ambient temperature, washed again, and examined by fluorescent microscopy. Samples were considered reactive when fluorescent bacterial morphology was evident. Samples reactive at the 1:80 screening dilution were considered positive and not titrated further, except for samples reactive with *A*. *phagocytophilum* and *E*. *chaffeensis* antigens that were serially titrated to an endpoint titer to exclude cross-reactivity between these species. Statistical analysis was performed by using the χ^2^ test to determine significant differences between groups. p values <0.05 were considered significant.

The median age of the 365 persons tested was 39 years (range 7–72 years) (Appendix Table 1) and the male:female sex ratio was 1.23 (205:160). All persons were engaged in livestock-rearing activities and spent substantial time outdoors. All 8 farms had pigs, sheep, horses, and cattle grazing on pastures. Among participants, 79.8% handled animals in pastures (graziers), 14.5% milked the animals (milkers), 2.9% worked in packing houses, 1.7% were veterinarians, and 1.2% assisted with animal birthing. The average length of service was 6.6 years (range 20 days to 45 years).

Appendix Table 2 shows the seroprevalence of infections by region and sex. Of 365 samples, 3 had insufficient volume for *A*. *phagocytophilum* testing. The highest *A*. *phagocytophilum* titer was 640; 32 (8.8%) had titers >80, 19 (5.3%) had titers >160, and 5 (1.4%) had titers >320. No significant differences between seroprevalence rates were found among the 8 communities surveyed (p>0.05). Tanggu (6/49, 12.2%) and Xiping (5/50, 10.0%) had the highest seroprevalence rates at a cutoff titer of 80; Xiping (5/50, 10.0%) and Hangu (4/45, 8.9%) had the highest seroprevalence rates at a cutoff titer of 160; and Xiping (2/50, 4.0%) had the highest seroprevalence rate at a cutoff titer of 320. Among 23 *A*. *phagocytophilum*–reactive sera tested for cross-reactivity, only 1 sample from Tanggu contained antibodies to *E*. *chaffeensis* at a titer of 160; this sample had an *A*. *phagocytophilum* titer of 80. Serologic analysis of 228 serum samples from all regions for *E*. *chaffeensis* showed no additional reactivity, with an overall seropositive rate of 0.4%.

A total of 220 serum samples showed seroprevalences of 9.5% (21/220) for *B*. *henselae*, 6.4% (14/220) for *C*. *burnetii*, and 4.1% (9/220) for *R*. *typhi*. The highest rates for *B*. *henselae* (22.9%) and *R*. *typhi* (18.8%) were found in Tanggu (both p<0.001); antibodies to *R*. *typhi* were not found in other locations. High seroprevalence rates for *B*. *henselae* were also identified in Xiqing (16.7%) and Jinnan (12.5%). The higher seroprevalence of *B*. *henselae* and *R*. *typhi* in Tanggu may be related to its low altitude, proximity to the Bo Sea (Bohai), or its port industry, which are environments conducive for fleas and their hosts (*8*). Antibodies to *C*. *burnetii* were found most often in Beichen (17.9%) and Xiqing (12.5%) (p<0.003), and seroprevalence was higher than that reported for the same area (*9*). Whether *C*. *burnetii* is an important pathogen in China needs further investigation.

There were no differences in seropositivity for antibodies to *A*. *phagocytophilum*, *B*. *henselae*, *R*. *typhi*, and *C*. *burnetii* by sex of the person tested. Seroprevalence of *A*. *phagocytophilum* was also similar across age groups, although the youngest group included all children <15 years of age, a potential bias given frequent exposure earlier in childhood (*10*). Antibodies to *B*. *henselae* and *R*. *typhi* were detected mainly in persons 20–50 years of age. Seroprevalence of *C*. *burnetii* was highest in persons 30–50 years of age. No differences in seroprevalence for any infections were found among graziers, milkers, packing house workers, veterinarians, or animal-birthing attendants, or among those with different lengths of farming service.

## Conclusions

Rickettsioses are zoonoses for which risk factors include exposure to vectors carrying the pathogens (*11*); human infections occur often where such exposures are frequent. The emerging pathogens *A*. *phagocytophilum* and *E*. *chaffeensis* are transmitted by tick vectors (*1*,*2*), and *R*. *typhi* is transmitted by fleas of rats or other reservoirs (*12*). Because of similar risk factors and ecologic conditions, infections with *Bartonella* spp. and *Coxiella* spp. were historically considered rickettsioses and are often examined together. However, *C*. *burnetii* is generally acquired by aerosols from parturient farm animals or wildlife or by ingestion of contaminated foods (*13*), and transmission of *Bartonella* spp. pathogenic for humans occurs through body lice for *B*. *quintana* and between pets by fleas and possibly ticks for *B*. *henselae* (*14*). Such ecologic and epidemiologic conditions are common in Tianjin (*9*).

Our results show that *A*. *phagocytophilum* and *B*. *henselae* are emerging and may already be established in Tianjin, with seroprevalences similar to those in North America and Europe (*15*). In contrast, there is little evidence to identify human *E*. *chaffeensis* infections. These findings support those of a study that showed that arthropod-transmitted rickettsiae, such as *R*. *typhi,* are prevalent in Tianjin and surrounding areas (*9*). Studies are needed to investigate these pathogens, their local vectors and reservoirs, and their role in the transmission of these agents. Such information would better define human infection risk and establish evidence for an etiologic differential diagnosis of febrile illnesses among people in these areas.

## Supplementary Material

Appendix Table 1 Seroprevalence of 5 bacterial zoonoses among farm workers, by age group, near Tianjin, People's Republic of China, May-July 2006*

Appendix Table 2 Seroprevalence of 5 bacterial zoonoses among farm workers in areas near Tianjin, People's Republic of China, May-July 2006*
