# Supplementary material for: Rickettsial Seroepidemiology among Farm Workers, Tianjin, People’s Republic of China
Source: Emerg Infect Dis. 2008 Jun;14(6):938–40. doi: 10.3201/eid1406.071502 (PMC2600283; doi:10.3201/eid1406.071502)
Supplement: Appendix Table 1 — Seroprevalence of 5 bacterial zoonoses among farm workers, by age group, near Tianjin, People's Republic of China, May-July 2006* [file 07-1502_appT1-s1.pdf]

Appendix Table 1. Seroprevalence of 5 bacterial zoonoses among farm workers, by age group, near Tianjin, People's Republic of China, May–July 2006*

Age, y	*Anaplasma phagocytophilum*			*Ehrlichia chaffeensis*			*Bartonella henselae*			*Coxiella burnetii*			*Rickettsia typhi*		
	M	F	Total	M	F	Total	M	F	Total	M	F	Total	M	F	Total
<15	14.3 (1/7)	0 (0/1)	12.5 (1/8)	0 (0/5)	NT	0 (0/5)	0 (0/7)	NT	0 (0/7)	0 (0/7)	NT	0 (0/7)	0 (0/7)	NT	0 (0/7)
15–19	12.5 (1/8)	11.1 (1/9)	11.8 (2/17)	0 (0/3)	0 (0/4)	0 (0/7)	0 (0/1)	0 (0/3)	0 (0/4)	0 (0/1)	0 (0/3)	0 (0/4)	0 (0/1)	0 (0/3)	0 (0/4)
20–29	15.8 (6/38)	3.5 (1/29)	10.5 (7/67)	0 (0/19)	0 (0/16)	0 (0/35)	5.6 (1/18)	20.0 (3/15)	12.1 (4/33)	0 (0/18)	0 (0/15)	0 (0/33)	5.6 (1/18)	0 (0/15)	3.0 (1/33)
30–39	5.7 (3/53)	2.3 (1/44)	4.1 (4/97)	0 (0/38)	0 (0/30)	0 (0/68)	7.9 (3/38)	12.9 (4/31)	10.1 (7/69)	2.6 (1/38)	9.7 (3/31)	5.8 (4/69)	5.3 (2/38)	6.5 (2/31)	5.8 (4/69)
40–49	10.5 (6/57)	10.0 (4/40)	10.3 (10/97)	0 (0/39)	3.2 (1/32)	1.4 (1/71)	16.7 (6/36)	6.5 (2/31)	11.9 (8/67)	16.7 (6/36)	9.7 (3/31)	13.4 (9/67)	5.6 (2/36)	3.2 (1/31)	4.5 (3/67)
50–59	12.5 (4/32)	10.0 (3/30)	11.3 (7/62)	0 (0/16)	0 (0/19)	0 (0/35)	7.1 (1/14)	5.3 (1/19)	6.1 (2/33)	0 (0/14)	5.3 (1/19)	3.0 (1/33)	0 (0/14)	5.3 (1/19)	3.0 (1/33)
≥60	11.1 (1/9)	0 (0/5)	7.1 (1/14)	0 (0/4)	0 (0/3)	0 (0/7)	0 (0/4)	0 (0/3)	0 (0/7)	0 (0/4)	0 (0/3)	0 (0/7)	0 (0/4)	0 (0/3)	0 (0/7)
Total	10.8 (22/204)	6.3 (10/158)	8.8 (32/362)	0 (0/124)	1.0 (1/104)	0.4 (1/228)	9.4 (11/118)	9.8 (10/102)	9.6 (21/220)	5.9 (7/118)	6.9 (7/102)	6.4 (14/220)	4.2 (5/118)	3.9 (4/102)	4.1 (9/220)

*Values are % (no. positive/no. tested); NT, none tested.
