# Supplementary material for: Rickettsial Seroepidemiology among Farm Workers, Tianjin, People’s Republic of China
Source: Emerg Infect Dis. 2008 Jun;14(6):938–40. doi: 10.3201/eid1406.071502 (PMC2600283; doi:10.3201/eid1406.071502)
Supplement: Appendix Table 2 — Seroprevalence of 5 bacterial zoonoses among farm workers in areas near Tianjin, People's Republic of China, May-July 2006* [file 07-1502_appT2-s2.pdf]

Appendix Table 2. Seroprevalence of 5 bacterial zoonoses among farm workers in areas near Tianjin, People's Republic of China, May–July 2006*

Area	*Anaplasma phagocytophilum*			*Ehrlichia chaffeensis*			*Bartonella henselae*			*Coxiella burnetii*			*Rickettsia typhi*		
	M	F	Total	M	F	Total	M	F	Total	M	F	Total	M	F	Total
Hangu	15.4 (4/26)	0 (0/19)	8.9 (4/45)	0 (0/12)	0 (0/13)	0 (0/25)	0 (0/17)	0 (0/7)	0 (0/24)	0 (0/17)	0 (0/7)	0 (0/24)	0 (0/17)	0 (0/7)	0 (0/24)
Dagang	3.6 (1/28)	10.0 (2/20)	6.3 (3/48)	0 (0/15)	0 (0/16)	0 (0/31)	10 (1/10)	0 (0/14)	4.2 (1/24)	0 (0/10)	7.1 (1/14)	4.2 (1/24)	0 (0/10)	0 (0/14)	0 (0/24)
Xiqing	7.7 (2/26)	12.5 (3/24)	10.0 (5/50)	0 (0/16)	0 (0/16)	0 (0/31)	26.7 (4/15)	0 (0/9)	16.7 (4/24)	0.20 (3/15)	0 (0/9)	12.5 (3/24)	0 (0/15)	0 (0/9)	0 (0/24)
Jinnan	9.5 (2/21)	8.0 (2/25)	8.7 (4/46)	0 (0/13)	0 (0/15)	0 (0/28)	12.5 (2/16)	12.5 (1/8)	12.5 (1/24)	0 (0/16)	0 (0/8)	0 (0/24)	0 (0/16)	0 (0/8)	0 (0/24)
Tanggu	13.8 (4/29)	10.0 (2/20)	12.2 (6/49)	0 (0/21)	0 (0/15)	0 (1/36)	17.6 (3/17)	25.8 (8/31)	22.9 (11/48)	0 (0/17)	9.7 (3/31)	6.3 (3/48)	29.4 (5/17)	12.9 (4/31)	18.8 (9/48)
Ninghe	8.3 (2/24)	0 (0/9)	6.1 (2/33)	0 (0/14)	0 (0/6)	0 (0/20)	7.1 (1/14)	0 (0/10)	4.2 (1/24)	7.1 (1/14)	10.0 (1/10)	8.3 (2/24)	0 (0/14)	0 (0/10)	0 (0/24)
Beichen	20.0 (4/20)	0 (0/25)	8.9 (4/45)	0 (0/18)	6.7 (1/15)	3.5 (1/32)	0 (0/14)	0 (0/14)	0 (0/28)	21.4 (3/14)	14.3 (2/14)	17.9 (5/28)	0 (0/14)	0 (0/14)	0 (0/28)
Dongli	20.7 (3/30)	6.3 (1/16)	8.7 (4/46)	0 (0/15)	0 (0/9)	0 (0/24)	0 (0/16)	12.5 (1/8)	4.2 (1/24)	0 (0/16)	0 (0/8)	0 (0/24)	0 (0/16)	0 (0/8)	0 (0/24)
Total	10.8 (22/203)	6.3 (10/158)	8.8 (32/362)	0 (0/124)	1.0 (1/104)	0.4 (1/228)	9.2 (11/119)	9.9 (10/101)	9.6 (21/220)	5.9 (7/119)	6.9 (7/101)	6.4 (14/220)	4.2 (5/119)	4.0 (4/101)	4.1 (9/220)

*Values are % (no. positive/no. tested).
